# Mitogenome-Informed Metabarcoding for Non-Invasive Identification of Sympatric Flamingos in Northern Chile

**DOI:** 10.3390/biology15151295

**Published:** 2026-08-04

**Authors:** Stephanie Barría-Iriarte, Diego Soto-Jiménez, Flavia Salcedo-Díaz, Noemí Labra-Oróstica, Pablo Aguilar, Pablo Valladares-Faúndez, Juan P. Muñoz, Claudio Quezada-Romegialli

**Affiliations:** 1 Plataforma de Monitoreo Genómico y Ambiental, Departamento de Química, Facultad de Ciencias, Universidad de Tarapacá, Arica 1000007, Chile; stephanie.barria2@gmail.com; 2 Centro Nacional de Investigación en Ríos, Invasiones y Sistemas (IRIS), Chile; 3 Laboratorio de Bioquímica, Departamento de Química, Facultad de Ciencias, Universidad de Tarapacá, Arica 1000007, Chile; diego.soto.jimenez@alumnos.uta.cl; 4 Unidad de Medio Ambiente del Consejo de Pueblos Atacameños (UMA-CPA), San Pedro de Atacama 1410000, Chile; flavia.salcedo.diaz@gmail.com (F.S.-D.); nlabraorostica@gmail.com (N.L.-O.); 5 Laboratorio de Complejidad Microbiana y Ecología Funcional, Instituto Antofagasta, and Centro de Bioingeniería y Biotecnología (CeBiB), Universidad de Antofagasta, Antofagasta 1240000, Chile; pablo.aguilar@uantof.cl; 6 Departamento de Biotecnología, Facultad de Ciencias del Mar y Recursos Biológicos, Universidad de Antofagasta, Antofagasta 1240000, Chile; 7 Departamento de Biología, Facultad de Ciencias, Universidad de Tarapacá, Arica 1000007, Chile; pvalladares@academicos.uta.cl

**Keywords:** *Phoenicopterus chilensis*, mitogenome, metabarcoding, environmental DNA, Bayesian inference, maximum likelihood, conservation genomics, Andean wetlands

## Abstract

The Chilean flamingo (*Phoenicopterus chilensis*) is a widely distributed South American species inhabiting diverse wetland ecosystems from Ecuador and Peru south to Chile and Argentina, and east to Brazil, Uruguay, and Paraguay, ranging from sea level to high-altitude Andean salars. Despite its broad distribution, local populations are increasingly exposed to anthropogenic pressures, raising conservation concerns at regional scales. In this study, we generated mitogenomic resources and developed a molecular framework for species identification using non-invasive samples. We sequenced genomic DNA from *P. chilensis* and assembled its complete mitogenome, enabling the in silico evaluation of commonly used metabarcoding markers. Subsequently, we collected eight fecal samples from a feeding site in a high-Andean lagoon where *P. chilensis* co-occurs with *Phoenicoparrus andinus* and *Phoenicoparrus jamesi*. As a positive control, nine additional fecal samples were collected from the Zoológico Nacional, Santiago, Chile, where only *P. chilensis* is maintained. DNA was extracted and initially screened via Sanger sequencing. To robustly assign taxonomic identity, we implemented a probabilistic framework combining maximum likelihood and Bayesian inference to estimate the posterior probability of species membership based on observed genetic variants. This approach revealed the presence of two genetically distinct groups not attributable to *P. chilensis*, indicating the coexistence and detectability of other flamingo taxa through fecal DNA. Our results demonstrate the value of integrating mitogenomic resources, metabarcoding, and statistical inference for non-invasive species identification in mixed-species systems. This framework provides a scalable tool for biodiversity monitoring and has direct implications for conservation strategies and evidence-based policy development in fragile high-Andean ecosystems.

## Simple Summary

Flamingos are important birds in South American wetlands, but distinguishing among species can be challenging where they occur sympatrically or when only fecal samples are available for analysis. This study developed a non-invasive method to identify flamingos from feces collected in northern Chile. To do this, the complete mitochondrial genetic information of the Chilean flamingo was generated and used as a reference to analyze short genetic fragments recovered from fecal samples. The method successfully identified samples associated with the Chilean flamingo and also detected two other genetically distinct flamingo lineages in natural wetland sites. These results show that fecal samples can provide reliable information about which flamingo species or lineages are using specific wetlands, without the need to capture or disturb the birds. This approach could be useful for monitoring flamingo populations, supporting conservation programs, and improving environmental management in fragile high-Andean wetlands.

## 1. Introduction

Flamingos represent one of the most emblematic groups of aquatic birds inhabiting South American wetlands and play an important ecological role as specialized consumers and visible indicators of environmental change [1,2]. In northern Chile and the Altiplano of the central Andes, at least three species may coexist, partially or locally: the Chilean flamingo (*Phoenicopterus chilensis*), the Andean flamingo (*Phoenicoparrus andinus*), and James’s flamingo (*Phoenicoparrus jamesi*) [3,4]. Regional studies on abundance and distribution have shown that a substantial proportion of these populations depends on high-Andean and Puna wetlands, highlighting the ecological importance of these systems for feeding, seasonal movements, and reproduction [4,5]. These wetlands are highly distinctive and productive ecosystems, but they are also fragile because they depend on hydrological balances that are sensitive to both climatic variability and anthropogenic disturbances [1,6].

In recent years, increasing concern has emerged regarding the combined effects of climate change, declining surface-water availability, and the expansion of lithium mining in the so-called “Lithium Triangle”, which includes areas of northern Chile [6,7]. Within this context, flamingos have been proposed as flagship species for the conservation of high-altitude wetlands, as their abundance and distribution respond to environmental processes operating at the landscape scale. Indeed, recent studies suggest that reductions in surface water and lithium-associated mining activity may negatively influence the abundance of some flamingo populations, reinforcing the need to develop robust monitoring tools for these ecosystems [7].

Despite their ecological and conservation relevance, species-level identification of flamingos in mixed flocks or from non-invasive samples can be challenging [4,8]. Field identification may be limited by the spatial coexistence of multiple species and by the logistical difficulties inherent to monitoring populations that occupy extensive, remote, and difficult-to-access wetlands. In addition, materials such as feces lack diagnostic morphological traits that allow unequivocal taxonomic assignment [8]. This methodological limitation is particularly relevant in protected wetlands or in areas highly sensitive to disturbance, where non-invasive sampling represents a valuable strategy. Although previous molecular studies have contributed to clarifying evolutionary relationships within *Phoenicopteridae*, available genomic reference resources for flamingos remain limited relative to the ecological and biogeographic importance of the group, particularly for South American taxa of Andean relevance [9,10]. In this scenario, complete mitochondrial genomes constitute a particularly useful resource because they provide high-resolution reference sequences for phylogenetic inference, marker evaluation, taxonomic assignment, and the interpretation of short amplicons generated through metabarcoding approaches [11]. Although complete mitogenomes have already been published for some members of the family, such as the American flamingo (*Phoenicopterus ruber*) [10], South American flamingos, particularly high-Andean taxa, remain underrepresented in terms of complete mitogenomic resources in the published literature and public repositories. This gap limits the accuracy of molecular identification tools in regions where several flamingo species may coexist.

DNA-based approaches offer a promising pathway to overcome this limitation. In recent years, the use of environmental DNA and metabarcoding for avian detection has increased considerably, and the MiBird primer set, a bird-specific metabarcoding primer set targeting a short fragment of the mitochondrial 12S rRNA gene, has proven useful for detecting and identifying birds from environmental samples when direct observation methods are incomplete or difficult to implement [12]. These strategies are especially attractive for non-invasive materials, such as feces, because they allow taxonomic information to be recovered from degraded DNA and can provide a more refined characterization of diet and consumed resources than approaches based exclusively on morphological analyses [13,14]. Moreover, a recent study conducted in hypersaline lakes of northern Chile applied a multimarker eDNA approach based on 12S, 18S rRNA, and cytochrome c oxidase subunit I (COI), showing that these methodologies can recover information from a broad range of taxa, including invertebrates and flamingos, and may therefore constitute useful tools for biomonitoring threatened saline wetlands [15].

The trophic dimension is particularly relevant because flamingo feeding ecology may vary among species and habitats [16]. In Chile, studies on *P. chilensis* have documented dietary composition through fecal analysis in coastal wetlands of Chiloé, including temporal variation in consumed items [17,18]. Comparative studies in South America have also reported differences in diet, feeding selectivity, and resource use among sympatric species, such as *P. andinus* and *P. chilensis* in lowland wintering areas [16]. Nevertheless, the available literature suggests that dietary studies in South American flamingos remain limited in both taxonomic and geographic coverage, and that molecular approaches to assess diet are still relatively scarce for these taxa [8,16]. In this context, an integrative framework combining mitogenomic reference resources, species assignment from fecal DNA, and dietary analysis through metabarcoding may contribute simultaneously to taxonomy, ecology, and conservation-oriented monitoring [10,12].

In the present study, the complete mitogenome of the *P. chilensis* was assembled and used as part of a molecular framework to analyze unidentified flamingo fecal samples collected in northern Chile. First, conventional Sanger sequencing was compared with a high-resolution metabarcoding approach based on mitochondrial primers. Subsequently, the generated data were evaluated to identify genetic clusters within the analyzed material, including one assignable to *P. chilensis*. Overall, this study provides a new genomic resource for South American flamingos and proposes a scalable framework for the non-invasive monitoring of cryptic genetic structure and trophic ecology in sympatric assemblages of aquatic birds.

## 2. Materials and Methods

### 2.1. Sampling of P. chilensis and Fecal Samples

Liver tissue samples from *P. chilensis* were obtained for genomic DNA extraction and subsequent genomic sequencing. This tissue was selected as the source of genomic DNA because it provides abundant host-derived cellular material while reducing the risk of contamination with dietary, microbial, or environmental DNA that may be present in other abdominal viscera. Tissue samples were collected in accordance with institutional and national regulations governing the use of animal-derived materials (authorized under Wildlife Movement Permit No. 23653, dated 19 June 2020 and managed by Pablo Valladares). Immediately after collection, samples were preserved in sterile tubes and stored at −80 °C until DNA extraction to ensure nucleic acid integrity. In addition, fecal samples were collected from two study sites in Chile: Laguna de Machuca, Antofagasta Region and Zoológico Nacional, located within the Parque Metropolitano de Santiago, Metropolitan Region. Fecal samples from Laguna de Machuca were collected on 4 August 2025, whereas samples from Zoológico Nacional were collected on 8 August 2025. The two collections were conducted on different dates because the sampling sites are geographically distant, approximately 1657 km apart (Table 1). Nevertheless, all fecal samples were collected as fresh/wet material and processed using the same laboratory workflow. Laguna de Machuca is a high-Andean wetland located within the Salar de Atacama basin in northern Chile, at approximately 4000 m a.s.l. This wetland represents an ecologically important habitat for the three flamingo species occurring in Chile: *P. chilensis* (Chilean flamingo), *P. andinus* (Andean flamingo), and *P. jamesi* (James’s flamingo). The site is used mainly as a feeding area and as a site for social and reproductive aggregation, including pair formation. Given its ecological relevance for these sympatric flamingo species, Laguna de Machuca was selected as a focal study site.

Sampling was conducted during daylight hours, prioritizing freshly deposited fecal material based on moisture, color, and absence of environmental degradation. Because moisture content and post-deposition exposure can influence DNA preservation, extraction yield, and amplification success, we selected fresh or moist fecal samples, which are more suitable for recovering host-associated DNA than dry or environmentally exposed samples, where DNA is expected to be more fragmented and to include a greater environmental or microbial contribution. Consequently, host taxonomic assignments were interpreted primarily from short mitochondrial amplicons and dominant ASV profiles, whereas microbial or dietary signals were considered more variable among samples and were interpreted cautiously.

Fecal samples were collected following non-invasive sampling criteria, avoiding direct interaction with individuals and minimizing disturbance to the animals. To reduce the risk of cross-contamination, each sample was handled independently using sterile gloves, disposable spatulas, and sterile collection tubes. Samples were immediately placed in sterile, pre-labeled containers and preserved either by refrigeration (4 °C) during field transport or in nucleic acid stabilization buffer when available. All samples were subsequently stored at −20 °C or −80 °C until laboratory processing.

Field metadata, including sampling location, date, fecal condition, and environmental conditions, were recorded for each sample. All procedures were designed to ensure sample traceability, reproducibility, and compliance with best practices for non-invasive genetic sampling.

### 2.2. DNA Extraction and Next- Generation Sequencing

For liver tissue samples of *P. chilensis*, a preliminary dissection was performed using a sterile scalpel under aseptic conditions to obtain a suitable tissue fragment for genomic DNA extraction (see Figure S1 for details). Then, genomic DNA was extracted using the DNeasy Blood & Tissue Kit (QIAGEN, Hilden, Germany), following the manufacturer’s instructions with minor adaptations to optimize tissue digestion. Briefly, liver tissue was placed in a sterile microcentrifuge tube, and the initial lysis step was performed by adding 180 µL of ATL buffer and 20 µL of proteinase K. The mixture was homogenized by vortexing and incubated at 56 °C for 6 h to promote tissue digestion and facilitate the release of genomic DNA. After enzymatic digestion, the extraction procedure was continued according to the manufacturer’s protocol, including DNA binding to the silica membrane, washing steps, and final elution.

The quality and integrity of the extracted DNA were evaluated by electrophoresis on a 1.8% agarose gel. For DNA visualization, 3 µL of SYBR Safe DNA Gel Stain (Invitrogen, Thermo Fisher Scientific, Waltham, MA, USA) was added to the gel. Electrophoresis was performed for 60 min at 90 V using a Model 200/2.0 Power Supply (Bio-Rad, Hercules, CA, USA). Each gel included 5 µL of AccuRuler 100 bp Plus DNA Ladder (MaestroGen, Hsinchu City, Taiwan) as a molecular size marker. For each sample, 5 µL of extracted DNA was mixed with 1 µL of loading buffer before loading onto the gel. DNA samples showing adequate integrity and quality were subsequently used for library preparation. Sequencing libraries were prepared using the MGIEasy Fast FS Library Prep Set V2.0 (MGI, Shenzhen, China), following the manufacturer’s protocol. Libraries were constructed through enzymatic fragmentation, adapter ligation, indexing, and amplification. High-throughput sequencing was performed using the DNBSEQ-G400RS High-throughput Sequencing Set on a DNBSEQ-G400 platform (MGI, Shenzhen, China), generating paired-end reads of 150 bp. All library preparation and sequencing procedures were conducted by TCL Genomics, Santiago, Chile.

### 2.3. Read Preprocessing, Mitochondrial Genome Assembly, and In Silico Primer Evaluation

Raw paired-end sequencing reads were subjected to a standardized preprocessing and quality control workflow implemented in custom Bash scripts. The workflow integrates adapter removal, quality trimming, and quantitative assessment of read retention and quality metrics. Raw FASTQ files (forward and reverse reads) were first evaluated using FastQC [19] to assess base quality distributions, GC content, sequence length profiles, and potential adapter contamination. This initial diagnostic step provided a baseline characterization of sequencing performance prior to filtering. Adapter trimming and quality filtering were performed using Cutadapt v.4.4 [20]. Reads were processed in paired-end mode with explicit specification of platform-specific adapter sequences (MGI adapters). Quality trimming was applied to both read ends using a Phred score threshold of Q20, and reads shorter than 50 bp after trimming were discarded. Filtering was implemented using the --pair-filter=any criterion, ensuring that read pairs were retained only if both mates satisfied the filtering thresholds. This conservative approach minimizes the propagation of low-quality or orphan reads into downstream analyses. The output of this step consisted of filtered paired FASTQ files and a detailed trimming report, including the number of processed read pairs, retained pairs, adapter occurrences, and base-level trimming statistics.

Filtered paired-end reads were assembled using a reference-guided and iterative mapping strategy in Geneious Prime 2026.0.2. Initially, reads were mapped against a mitochondrial reference genome (*Phoenicopterus ruber*, Genbank accesion NC_027934 [10]) using the built-in mapper implemented in Geneious. Read mapping was performed using the Geneious alignment algorithm under a medium–low sensitivity/fast setting, prioritizing computational efficiency while maintaining adequate alignment stringency for closely related reference sequences. Structural variation detection was enabled, including identification of short insertions and deletions, with a maximum indel size threshold of 1000 bp. No additional fine-tuning parameters were applied beyond the default fast read-mapping configuration. Prior to mapping, reads were trimmed within Geneious to ensure consistency with internal alignment parameters. The assembly output included mapped reads (with paired-end information retained), contigs, and consensus sequences. The preliminary consensus was manually inspected, with particular attention to the control region. Because ambiguous bases were detected in some sectors of this region, additional targeted mappings were performed using BBMap v38.84 implemented in Geneious Prime under high sensitivity/slow settings. For these analyses, control-region reference fragments including the control region and adjacent mitochondrial genes were extracted from *Phoenicopterus roseus* (Genbank accesion NC_010089 [21]) and *P. ruber* (Genbank accesion NC_027934 [10]. Reads recruited in these targeted mappings were then remapped iteratively against the preliminary mitochondrial consensus to generate the final consensus mitogenome. Because the assembly was based on reference-guided mapping and iterative remapping rather than a de novo k-mer-based assembler, no user-defined k-mer parameter was applied. Assembly reports were generated to document mapping performance and alignment statistics. Final consensus mitochondrial sequences were exported for downstream analyses, including coverage assessment, variant evaluation, annotation, and GenBank submission.

The assembled and annotated mitochondrial genome of *P. chilensis* was used as a reference template to evaluate, in silico, binding position and expected amplicon size of two metabarcoding primer sets targeting mitochondrial ribosomal genes. MarVer3 primer pair of Valsecchi et al., (2020) [22], designed to amplify a variable region of the mitochondrial 16S rRNA gene, was evaluated using the sequences MarVer3F 5′-AGACGAGAAGACCCTRTG-3′ and MarVer3R 5′-GGATTGCGCTGTTATCCC-3′. This primer set was originally reported to produce amplicons of approximately 232–274 bp across marine vertebrates, with an average size of approximately 245 bp. The MiBird-U primer pair of Ushio et al. (2018) [12], designed for avian eDNA metabarcoding and targeting the mitochondrial 12S rRNA gene, was also evaluated using the primer sequences MiBird-U-F 5′-GGGGTTGGTAAATCTTGTGCCAGC-3′ and MiBird-U-R 5′-CATAGTGGGGTATCTAATCCCAGTTTG-3′. This marker corresponds to a short 12S fragment, originally reported with an insert length of approximately 171 bp.

Primer sequences were imported into Geneious Prime and mapped against the assembled mitochondrial genome using the corresponding IUPAC ambiguity codes. For each primer pair, the location of forward and reverse primer binding sites, strand orientation, number and position of mismatches, and predicted amplicon length were inspected manually. The amplified regions were then compared with the mitochondrial genome annotation to confirm that MarVer3 targeted the 16S rRNA gene and MiBird-U targeted the 12S rRNA gene. Predicted amplicons were extracted from the reference mitogenome and retained for downstream evaluation of primer suitability, expected taxonomic informativeness, and compatibility with short-read metabarcoding assays.

### 2.4. Sanger Sequencing Analysis of the Mitochondrial 12S Region

*Phoenicopterus chilensis* tissue samples were analyzed by Sanger sequencing to evaluate the amplification of informative mitochondrial regions suitable for molecular identification. For this purpose, primers associated with the MiBird metabarcoding system were used to target a fragment of the mitochondrial 12S ribosomal RNA gene. The primer sequences used for amplification are listed in Table 2.

The forward primer corresponded to MiBird-U-F, whereas the reverse primer corresponded to MiBird-U-R. This strategy was used to assess whether the selected 12S marker could generate sequence information suitable for downstream molecular discrimination of flamingo-derived DNA.

PCR amplification of the 12S mitochondrial marker was directly evaluated using DNA extracted from *P. chilensis* fecal samples (see the next section for fecal DNA extraction). This approach was selected to assess marker performance under the same non-invasive sample conditions intended for downstream molecular identification. Amplified products were inspected prior to sequencing to confirm the presence of bands within the expected size range and to exclude non-specific amplification. PCR products were then submitted to Macrogen Inc. (Santiago, Chile) for purification and Sanger sequencing. Sequence quality was inspected, and high-quality reads were used to evaluate the suitability of the MiBird 12S region for flamingo identification from non-invasive material.

### 2.5. Fecal DNA Extraction and High-Throughput Sequencing

Fecal samples collected from Laguna de Machuca (LM) and the Zoológico Nacional (ZN) were processed for fecal DNA extraction. Samples obtained from ZN were used as positive controls for *P. chilensis*, allowing the evaluation of amplification efficiency and the consistency of molecular taxonomic assignment.

Fecal DNA was extracted using the QIAamp^®^ PowerFecal^®^ Pro DNA Kit (QIAGEN, Hilden, Germany), following the manufacturer’s instructions. Because fecal samples may contain degraded DNA, polymerase chain reaction inhibitors, and genetic material from multiple organisms, strict handling procedures were implemented to reduce the risk of cross-contamination. Each sample was processed independently using sterile materials, and negative extraction controls were included when appropriate to monitor possible contamination during the extraction workflow.

The quality and integrity of the extracted DNA were evaluated by electrophoresis on a 1.8% agarose gel. For DNA visualization, 3 µL of SYBR Safe DNA Gel Stain (Invitrogen, Thermo Fisher Scientific, Waltham, MA, USA) was added to the gel. Electrophoresis was performed for 60 min at 90 V using a Model 200/2.0 Power Supply (Bio-Rad, Hercules, CA, USA). Each gel included 5 µL of AccuRuler 100 bp Plus DNA Ladder (MaestroGen, Hsinchu City, Taiwan) as a molecular size marker. For each sample, 5 µL of extracted DNA was mixed with 1 µL of loading buffer before loading onto the gel. After integrity assessment, fecal DNA was quantified using a DeNovix QFX Fluorometer (DeNovix, Wilmington, DE, USA) to estimate DNA concentration prior to library preparation. In addition, fragment quality and size distribution were evaluated using the Qsep system (BiOptic Inc., New Taipei City, Taiwan). These quality control steps were performed to determine whether the extracted DNA was suitable for downstream library construction and high-throughput sequencing.

Samples that met the minimum quality requirements were processed using a nested PCR-based amplicon library preparation workflow. The first PCR selectively amplified the mitochondrial 12S and 16S target regions from fecal DNA extracts using MiBird and MarVer3 primer sets respectively, whereas the second PCR incorporated sequencing adapters and sample-specific indices required for multiplexed sequencing. Amplicon libraries were purified, inspected to confirm the expected fragment size range, pooled, and sequenced in house on an Illumina iSeq 100 System (Illumina, San Diego, CA, USA). This workflow enabled high-throughput molecular identification of flamingo-derived DNA from non-invasive fecal samples.

### 2.6. Bioinformatic Processing of 16S rRNA Metabarcoding Data Using MarVer3 Primers

Raw paired-end Illumina reads generated from fecal DNA extracts amplified with the mitochondrial 12S (MiBird) and 16S (MarVer3) rRNA primers set [22] were processed in R using the DADA2 package [23], following an amplicon sequence variant (ASV)-based workflow. This denoising approach was selected because it infers exact biological sequence variants while explicitly modeling sequencing errors, thereby providing higher sequence resolution than traditional operational taxonomic unit clustering.

Forward and reverse FASTQ files were imported from a dedicated directory containing quality-controlled, demultiplexed reads. Sample identities were parsed directly from file names, and paired-end reads were matched according to the conventional _R1 and _R2 nomenclature. Initial read quality was assessed by visual inspection of per-base quality score profiles from representative forward and reverse libraries.

Reads were filtered and trimmed using the filterAndTrim() function. A minimum post-filter length of 100 bp was required. To remove primer sequences, barcode regions, and low-quality leading bases, the first 32 nucleotides of both forward and reverse reads were trimmed. This trimming threshold corresponded to the combined length of the MarVer3/MiBird primer sequences and inline barcode regions used during library preparation. Reads containing ambiguous bases were discarded (maxN = 0). Expected error thresholds were conservatively set to a maximum of two expected errors per read (maxEE = c(2,2)), and reads were truncated at the first base with a quality score ≤ 2 (truncQ = 2). PhiX control reads were removed in silico, and the filtered reads were retained as compressed FASTQ files.

Filtered reads were dereplicated independently for the forward and reverse directions using derepFastq(), collapsing identical reads into unique abundance-weighted sequences. Error rates were then empirically learned from the dataset using learnErrors(), allowing run-specific substitution patterns to be estimated rather than applying fixed error assumptions. The observed and estimated error profiles were inspected graphically to verify adequate model fit.

Exact sequence inference was performed separately for forward and reverse reads using the core dada() algorithm in pseudo-pooling mode. This strategy improves the recovery of low-abundance true variants shared across samples while maintaining greater computational efficiency than full pooling. Denoised forward and reverse reads were subsequently merged using mergePairs(), requiring sufficient and concordant overlap between paired reads. Read pairs with inconsistent assemblies were excluded from downstream analyses.

A sequence table containing per-sample ASV abundances was generated using makeSequenceTable(). Amplicon length distributions were inspected both numerically and graphically to verify consistency with the expected MarVer3/MiBird fragment size and to identify potential non-specific amplification products. Putative chimeric sequences were detected and removed de novo using removeBimeraDenovo() with the consensus method, which identifies variants classified as chimeric across multiple samples. The proportion of retained non-chimeric reads was calculated as an additional quality-control metric.

Read retention was summarized for each sample across all major processing steps, including: (i) raw input reads, (ii) filtered reads, (iii) denoised forward reads, (iv) denoised reverse reads, (v) merged read pairs, and (vi) final non-chimeric reads. This tracking step allowed the identification of libraries showing abnormal read loss, poor merging efficiency, or potential amplification artifacts.

Taxonomic assignment of non-chimeric ASVs was performed using assignTaxonomy() function against a curated local reference database. A minimum bootstrap confidence threshold of 90% was applied to reduce false-positive assignments and prioritize robust taxonomic classifications. This stringent threshold was particularly relevant for the identification of host-associated sequences and for the characterization of dietary components detected in flamingo fecal samples.

For downstream ecological and comparative analyses, the ASV abundance matrix, taxonomy table, and sample metadata were integrated into a phyloseq object. Sample metadata included collection site information extracted from file names. This integrated data structure facilitated subsequent analyses of taxonomic composition, relative abundance patterns, sample clustering, prey composition, and comparisons among fecal samples assigned to different flamingo genetic groups.

#### ASV Clustering Tree

Flamingo-associated ASV sequences were aligned in Geneious Prime 2026.0.2 using MUSCLE [24] with default parameters. The resulting alignment was used to infer an approximately maximum-likelihood tree in Geneious Prime using FastTree v.2.1.11 [25]. Tree inference was performed under the generalized time-reversible nucleotide substitution model, with 20 rate categories across sites and Gamma20 likelihood optimization. Branch support values correspond to FastTree SH-like local support. The resulting tree was interpreted as a sequence-similarity clustering of ASVs for lineage-level visualization, not as a formal species-level phylogenetic reconstruction.

### 2.7. Hierarchical Assignment of Species Identity and Intraspecific Genetic Variants from Amplicon Sequence Variants

To infer flamingo taxonomic identity from fecal DNA-derived amplicon sequence variants (ASVs), we implemented a hierarchical probabilistic framework that separates species-level assignment from within-species variant composition. This approach was adopted because multiple recurrent ASVs were detected within each focal lineage, potentially reflecting true mitochondrial polymorphism, geographically structured haplotypes, residual sequencing artifacts, or rare biological variants.

After denoising and ASV curation, biologically interpretable variants were grouped into three higher-order taxonomic units based on sequence similarity, phylogenetic affinity, and reference comparisons: *P. chilensis*, Parina lineage A, and Parina lineage B. Because reference databases remain incomplete for Andean and James’s flamingos, the two Parina lineages were conservatively retained as independent operational species-level units pending definitive attribution to *P. andinus* and *P. jamesi*.

For each sample *j*, let *x_jk_* denote the number of reads assigned to ASV *k*, and let the total number of informative reads in that sample be:

(1) $$N_{j}=\sum_{k=1}^{K}x_{jk}$$

The vector of observed ASV counts for sample j is therefore:

(2) $$x_{j}=\left(x_{j1},x_{j2},\ldots ,x_{jK}\right)$$

Observed counts were modelled as a multinomial sampling process

(3) $$x_{j}∼Multinomial\left(N_{j},\pi_{j}\right)$$

where $\pi_{j}$ is the vector of ASV probabilities in sample j, representing the expected read proportions generated by the combined effects of species identity and within-species variant composition. Because ASVs were nested within species groups, the probability of observing a given variant was modelled hierarchically as the product of a species-level component and a within-species component; in conceptual terms,

(4) $$P({ASV}_{k})=P({Species}_{s})\times P({ASV}_{k}|{Species}_{s})$$

for ${ASV}_{k}$ belonging to species group s. Accordingly, the expected probability of observing ASV k in sample j was parameterized as:

(5) $$\pi_{jk}=p_{js}q_{k|s}$$

where $p_{js}$ denotes the probability that a read from sample j belongs to species group s, and $q_{k|s}$ denotes the conditional relative frequency of ASV k within that species group. For each sample, reads assigned to all ASVs belonging to the same species group were aggregated as:

(6) $$Y_{js}=\;\sum_{k\in s}x_{jk}$$

where $Y_{j1}$, $Y_{j2}$ and $Y_{j3}$ correspond to the total reads assigned to *P. chilensis*, Parina lineage A, and Parina lineage B, respectively. The total informative read count across species groups is therefore given by:

(7) $$N_{j}=\;\sum_{s=1}^{3}x_{jk}$$

Species-level probabilities were first estimated under the multinomial model using the maximum-likelihood estimator (MLE):

(8) $${\hat{p}}_{js}^{MLE}=\frac{Y_{js}}{N_{j}}$$

This estimator corresponds to the observed proportion of reads assigned to species groups in sample *j*. The dominant group was initially identified as the unit with the highest estimated probability, whereas final assignments were subsequently evaluated using predefined confidence thresholds. To stabilize estimates in low-depth samples, reduce sensitivity to rare carryover reads, and avoid zero estimates, Bayesian posterior probabilities were additionally calculated under a conjugate Dirichlet-multinomial framework. Assuming a Dirichlet prior,

(9) $$p_{j}∼Dirichlet\left(\alpha_{j1},\alpha_{j2},\alpha_{j3}\right)$$

where $\alpha_{js}$ are prior concentration parameters. When no prior ecological information was available, a uniform prior (1,1,1) was used, assigning equal prior support to all species groups. When available, informative priors could be specified globally or by locality, reflecting known flamingo assemblages. Given observed grouped counts $Y_{js}$, posterior mean probabilities were then calculated as:

(10) $${\hat{p}}_{js}^{\;Bayes}=\frac{Y_{js}+\;\alpha_{js}}{N_{j}+\;\sum_{s=1}^{3}\alpha_{js}}$$

These posterior means were used as shrinkage estimators robust to rare carryover reads, tag-jumping and stochastic low-frequency observations.

Final species assignments were based on posterior probabilities, with *MLE* estimates retained for comparison. Samples were conservatively assigned to species group s when:

(11) $${\hat{p}}_{js}\geq 0.95$$

where ${\hat{p}}_{js}$ denotes the selected species-level probability estimate. Samples failing this criterion were classified as mixed when two groups showed substantial support, ambiguous when no clear dominant group was present, or low-information when total read counts were insufficient for reliable inference.

Edge cases were explicitly considered in the interpretation of the assignment framework. Samples with very low numbers of informative reads were treated as low-information unless the dominant lineage was supported by sufficient read depth and, when available, replicate consistency. Likewise, samples in which the dominant lineage only marginally exceeded the 0.95 threshold were regarded as borderline assignments rather than as equivalent to samples showing near-complete dominance of a single lineage. In such cases, final interpretation considered the total number of informative reads, the absolute and relative abundance of secondary lineages, negative controls, possible index hopping or tag-jumping, and the ecological or geographic plausibility of the assignment. To further reduce the influence of trace-level signals, optional abundance filtering was considered before final interpretation. Such filtering can be defined either at the dataset level, based on the total abundance of each ASV or lineage across all samples, or at the sample level, based on its proportional contribution within each individual sample. For species assignment, sample-level filtering is generally more informative because the same relative abundance may represent very different absolute read counts depending on sequencing depth. For example, 1% of 100,000 informative reads represents 1000 reads, whereas 1% of 2500 reads represents only 25 reads. Therefore, low-frequency secondary lineages were evaluated using both relative abundance and absolute read count criteria. In conservative single-host assignment, secondary lineage signals below 1–3% of informative reads within a sample, particularly when represented by low absolute counts and not supported by replicate consistency, were flagged as probable background rather than interpreted as evidence of mixed origin. A more stringent threshold, such as 5%, may be appropriate when the analytical objective is to minimize false mixed assignments caused by low-level carryover, index hopping, or tag-jumping.

For confidently assigned samples, within-species ASV composition was estimated as:

(12) $$q_{jk|s}=\frac{x_{jk}}{Y_{js}}$$

where $q_{jk|s}$ represents the relative contribution of ASV k within species group s. This allowed distinction between dominant haplotypes and rare internal variants.

Very low-frequency ASVs were retained in the dataset but were not used alone to overturn strong species-level assignments. Such reads may reflect residual PCR or sequencing artifacts, index hopping or tag-jumping, environmental trace DNA, cross-sample carryover, or genuine minor haplotypes. All calculations were implemented in R v4.5.1 [26] and Rstudio v2025.09.2.418 [27] using custom scripts.

## 3. Results

### 3.1. Assembly of the P. chilensis Mitogenome and In Silico Evaluation of the MarVer3 Marker

**Figure 1:**
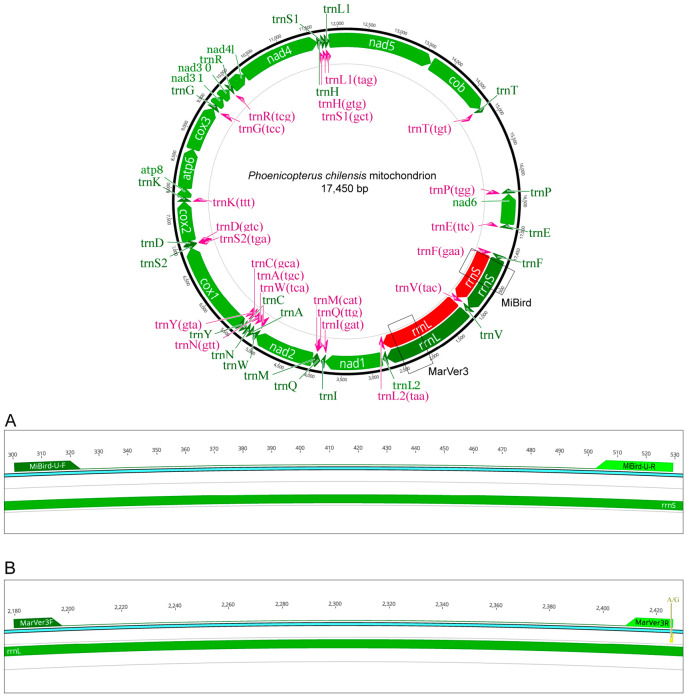
Complete mitochondrial genome of *Phoenicopterus chilensis* and localization of metabarcoding primer-binding regions. Circular representation of the complete mitochondrial genome of *P. chilensis*. The assembled mitogenome comprised 17,450 bp. Protein-coding genes and associated gene features are shown in green, ribosomal RNA genes (rrnS and rrnL) in red, and transfer RNA genes in magenta, with their corresponding anticodons indicated in parentheses. Arrows indicate the direction of transcription. (**A**) Localization of the MiBird-U-F and MiBird-U-R primer-binding sites within the mitochondrial 12S rRNA region. (**B**) Localization of the MarVer3F and MarVer3R primer-binding sites within the mitochondrial 16S rRNA region.

High-throughput sequencing of genomic DNA obtained from *P. chilensis* tissue generated 6,788,954 paired-end reads using the DNBSEQ-G400RS High-throughput Sequencing Set in paired-end 150 bp mode. These reads enabled the reconstruction of the complete mitochondrial genome of this species. The assembled circular mitogenome had an ungapped consensus length of 17,450 bp (Genbank accession number PZ609876), providing a species-specific reference resource for downstream molecular identification of flamingo-derived DNA from non-invasive samples. Read mapping covered 100% of the mitochondrial reference length, with a mean sequencing depth of 50.9×, a standard deviation of 34.1×, and a coverage range of 3× to 333×. Forward and reverse strand coverage were balanced, with mean values of 25.9× and 25.0×, respectively. The final consensus contained two ambiguous N positions, corresponding to low-confidence sites in the control region. The circular representation of the mitogenome showed the expected mitochondrial structure and allowed the localization of the MarVer3F/MarVer3R and MiBirdU-F/MiBird-U-R primers-binding region, supporting its suitability for amplification of a short mitochondrial fragment compatible with degraded DNA obtained from fecal material (Figure 1).

The in silico evaluation of the MarVer3 primer pair showed that the amplified region was located within the mitochondrial genome and contained informative sequence variation useful for discriminating among flamingo-associated amplicon sequence variants. This reference-guided strategy was therefore used to support subsequent metabarcoding-based identification of fecal samples collected from natural and controlled localities.

### 3.2. Amplicon Sequence Variant Profiles Reveal Three Main Flamingo Genetic Groups

Metabarcoding analysis of fecal DNA generated a set of flamingo-associated amplicon sequence variants (ASVs) that grouped into three major genetic units: *Phoenicopterus*, Parina lineage A, and Parina lineage B. The ASV abundance matrix showed a highly structured distribution across samples, with most samples dominated by a single ASV group and only minimal read contribution from secondary lineages (Table 3).

The *Phoenicopterus* group was detected almost exclusively in samples from the Zoológico Nacional. Within this group, *Phoenicopterus*_01 was the dominant ASV in most ZN samples, with high read counts in ZN-F01, ZN-F02, ZN-F03, ZN-F06, ZN-F07, ZN-F09, ZN-F11, and ZN-F12. A second variant, *Phoenicopterus*_02, was detected only in ZN-F10, where it represented the dominant *Phoenicopterus*-associated sequence. This pattern suggests the presence of at least two closely related mitochondrial variants within the *Phoenicopterus* group, while maintaining a consistent species-level signal across the ZN samples. In contrast, Parina lineage A was the dominant signal in samples from Laguna de Machuca. The ASV ParinaA_01 accounted for nearly all reads assigned to this lineage in LM-F01, LM-F02, LM-F04, LM-F05, and LM-F06. Although additional ASVs were included in the Parina A group, they were not represented by appreciable read counts in the analyzed dataset, indicating that ParinaA_01 was the major detectable variant under the conditions evaluated. Parina lineage B was mainly represented by two ASVs. ParinaB_02 was highly abundant in LM-F03, whereas ParinaB_01 dominated LM-F07 and LM-F08. This distribution indicates the presence of two distinct Parina B-associated mitochondrial variants among samples from Laguna de Machuca. Low numbers of Parina B reads were also detected in a small number of otherwise non-Parina B samples, but these signals were minor relative to the dominant lineage-specific read counts.

### 3.3. Genetic Clustering Supports the Separation of Phoenicopterus and Two Parina Lineages

**Figure 2:**
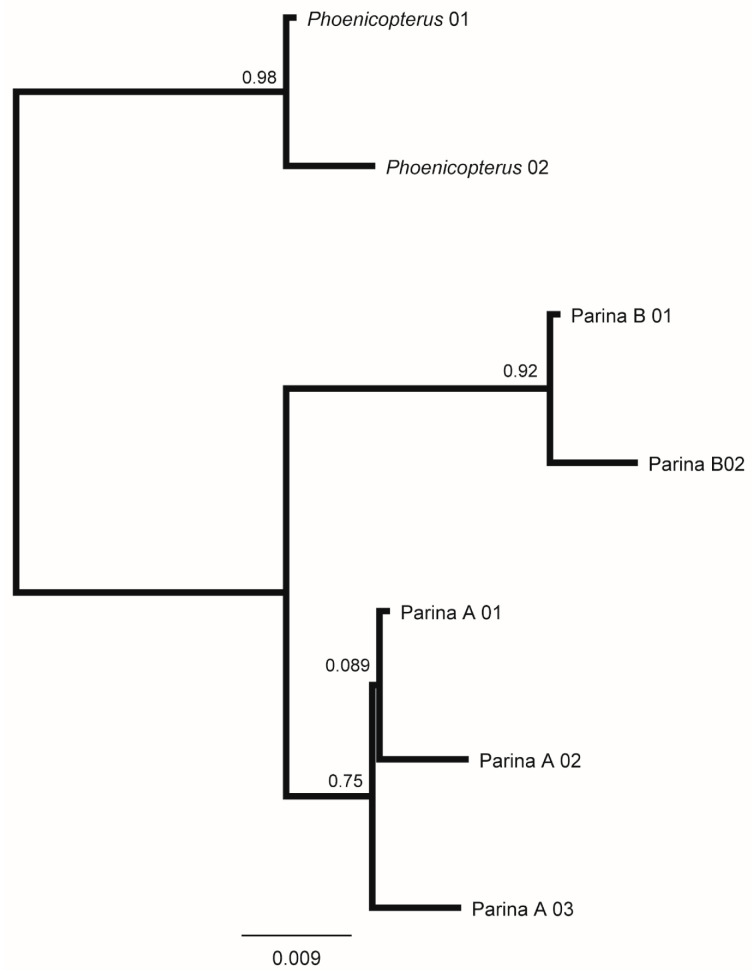
Tree inferred from aligned ASV sequences recovered from fecal metabarcoding. Branch support correspond to FastTree SH-like local support values. The tree separates the ASVs into three major groups: *Phoenicopterus*, Parina lineage A, and Parina lineage B.

The approximately maximum-likelihood tree inferred from the aligned flamingo-associated ASVs supported the separation of the dataset into three well-defined genetic clusters: *Phoenicopterus*, Parina A and Parina B (Figure 2). The two *Phoenicopterus* ASVs clustered together and were clearly separated from the Parina-associated ASVs. Within the Parina group, the analysis further distinguished Parina lineage A from Parina lineage B, supporting the presence of at least two genetically differentiated Parina-associated lineages in the analyzed fecal material.

The topology showed that ParinaB_01 and ParinaB_02 formed a distinct cluster, whereas ParinaA_01, ParinaA_02, and ParinaA_03 clustered within a separate Parina A branch. This pattern is consistent with the ASV abundance profiles and indicates that the recovered sequence variants were not randomly distributed among samples, but instead corresponded to discrete genetic units. Importantly, the clear separation between the *Phoenicopterus* and Parina-associated clusters supports the capacity of the selected mitochondrial metabarcoding approach to distinguish Chilean flamingo-derived fecal DNA from fecal material associated with Andean flamingo and James’s flamingo lineages.

### 3.4. Hierarchical Probabilistic Assignment Confirms Robust Species-Level Classification of Fecal Samples

A hierarchical probabilistic framework was applied to assign each fecal sample to one of the three genetic groups detected in the ASV analysis. Both the maximum likelihood estimator and the Bayesian Dirichlet model produced concordant classifications for all samples. In every case, the dominant genetic group exceeded the predefined confidence threshold for assignment, and no sample was classified as mixed, ambiguous, or low-information (Table 4). Although all samples exceeded the predefined 0.95 assignment threshold, two samples represented the closest cases to the decision boundary. VM-F05 was assigned to Parina lineage A with a Bayesian posterior probability of 0.994 and a minor Parina B signal of 0.006, whereas ZM-F12 was assigned to *Phoenicopterus* with a posterior probability of 0.994 and a minor Parina B signal of 0.006. These secondary components were below conservative sample-level abundance thresholds commonly used to flag trace-level background signals and did not alter the final assignments.

Samples from Laguna de Machuca showed a more heterogeneous pattern. LM-F01, LM-F02, LM-F04, LM-F05, and LM-F06 were assigned to Parina lineage A, whereas LM-F03, LM-F07, and LM-F08 were assigned to Parina lineage B. The assignment of LM-F03 to Parina B was particularly strong, with both maximum likelihood and Bayesian estimates reaching 1.000. Similarly, LM-F07 and LM-F08 showed Bayesian posterior probabilities of 0.998 and 0.997 for Parina B, respectively. These results indicate the co-occurrence of two genetically distinct Parina lineages at Laguna de Machuca.

All samples from the Zoológico Nacional locality were assigned to *Phoenicopterus*, consistent with their expected origin as *P. chilensis*-associated samples. Most ZN samples showed probabilities of 0.999–1.000 under the Bayesian model. ZN-F12 showed a dominant *Phoenicopterus* assignment with a posterior probability of 0.994, together with a minor Parina B signal of 0.006. This secondary signal was too low to alter the final taxonomic assignment.

### 3.5. Minor Secondary Signals Were Detected but Did Not Affect Final Assignments

Although all fecal samples were confidently assigned to a single dominant lineage, a limited number of samples showed trace-level reads associated with secondary groups. For example, LM-F05 was assigned to Parina lineage A but contained a minor Parina B component, whereas ZN-F12 was assigned to *Phoenicopterus* but showed a low Parina B contribution. These minor signals represented only a small fraction of the total reads and were far below the threshold required to classify a sample as mixed.

Given their low relative abundance, these secondary signals are most consistent with technical or environmental background sources, such as index hopping, low-level carryover, residual environmental DNA, or sequencing noise. At the sample level, these minor secondary signals represented less than 1% of informative reads. Therefore, they fell below even the least stringent conservative filtering criterion considered here and were interpreted as trace-level background rather than as evidence of mixed fecal origin. Importantly, the Bayesian Dirichlet model provided conservative posterior estimates while maintaining the same final assignments as the maximum likelihood approach, reinforcing the robustness of the classification framework.

### 3.6. Spatial Distribution of Flamingo Genetic Groups Across Sampling Localities

The assignment patterns revealed a clear spatial structure among the analyzed fecal samples. The Zoológico Nacional samples were exclusively associated with *Phoenicopterus*, validating the ability of the workflow to recover the expected *P. chilensis* signal from fecal DNA.

The Laguna de Machuca locality showed the highest genetic heterogeneity, with samples assigned to both Parina lineage A and Parina lineage B. This finding indicates that at least two genetically distinct Parina-associated lineages used this site during the sampling period. Therefore, the combination of non-invasive fecal sampling, mitochondrial metabarcoding, ASV-level resolution, and probabilistic classification enabled the detection of sympatric or locally co-occurring flamingo lineages that would be difficult to discriminate from fecal material by morphological criteria alone.

Collectively, these results demonstrate that mitogenome-informed metabarcoding provides a robust framework for the non-invasive molecular identification of flamingos in mixed-species systems. The approach not only confirmed the detection of *P. chilensis* in control samples but also revealed two additional Parina-associated genetic groups in northern Chilean wetland samples, highlighting its potential application for biodiversity monitoring and conservation-oriented surveillance in high-Andean aquatic ecosystems.

## 4. Discussion

This study provides a mitogenome-informed metabarcoding framework for the non-invasive molecular identification of flamingo fecal samples collected in northern Chile. By integrating the newly assembled *P. chilensis* mitochondrial genome with in silico primer evaluation, high-throughput sequencing, ASV-level resolution, and probabilistic taxonomic assignment, the results show that fecal DNA can discriminate *Phoenicopterus* from two genetically differentiated Parina-associated lineages. This is particularly relevant in high-Andean wetlands, where different flamingo species may coexist and fecal samples lack diagnostic morphological features for reliable taxonomic identification.

The complete mitochondrial genome of *P. chilensis* represents an important genomic resource for South American flamingos. Mitogenomes provide reference sequences for marker evaluation, phylogenetic inference, taxonomic assignment, and interpretation of short amplicons recovered from degraded biological material [28,29]. Although mitochondrial genomes have been reported for some flamingo species, including the American flamingo *P. ruber* [10] and the greater flamingo [21], mitogenomic resources for South American flamingos remain limited relative to their ecological and conservation importance. This reference gap is especially relevant in regions where *P. chilensis*, *P. andinus*, and *P. jamesi* may occur in sympatry. In this context, the *P. chilensis* mitogenome assembled here partially fills this gap and provides a species-specific mitochondrial reference for interpreting fecal metabarcoding data from South American flamingo assemblages.

The availability of this mitogenome also supports a conservative interpretation of fecal ASVs. Sequences matching *P. chilensis* can be interpreted against a species-specific mitochondrial reference, whereas ASVs that do not match *P. chilensis* can be retained as operational Parina lineages until complete and validated reference sequences for *P. andinus* and *P. jamesi* become available. This conservative treatment avoids assigning sequences beyond the current resolution of available reference databases, while still allowing biologically meaningful discrimination among flamingo-associated lineages. Future expansion in mitochondrial and metabarcoding reference databases for *P. andinus* and *P. jamesi* should refine the operational Parina lineages recovered here and may allow their assignment to named species with greater confidence. This improvement would increase the applicability of the proposed framework by enabling greater taxonomic precision on flamingo assemblages and supporting future studies of diet and trophic ecology through fecal metabarcoding. Similar improvements in taxonomic resolution have been observed as reference databases become more comprehensive and representative of natural genetic diversity [30,31]. Such improvements would also broaden the applicability of this framework beyond host identification, supporting future studies of flamingo assemblage composition, diet, trophic interactions, habitat use, and resource partitioning through fecal DNA metabarcoding [32,33].

The performance of metabarcoding primers depends on both theoretical compatibility with target sequences and empirical performance in complex biological samples [34]. In silico analyses allow primer-binding sites, expected amplicon position, and potential taxonomic resolution to be evaluated before sequencing; however, they cannot fully predict amplification success in complex samples such as feces [35]. The present study addresses this limitation by combining mitogenome-based primer localization with experimental validation and NGS-based application. The consistent assignment of Zoológico Nacional samples to *Phoenicopterus* further supports the practical utility of the marker for recovering host-associated DNA from fecal material. By locating the MarVer3 primer-binding region within the mitochondrial genome, this workflow directly connects the genomic reference with the downstream interpretation of fecal DNA sequencing data. Although the MarVer3 primer set was not specifically designed for flamingos, its binding to the *P. chilensis* mitochondrial genome was evaluated in silico, and only one mismatch at the 5′ end was detected for this lineage. This observation does not suggest a major primer-binding limitation for *P. chilensis*. Nevertheless, differential amplification efficiency among flamingo lineages cannot be completely excluded, because primer-template mismatches can influence amplification success and affect the relative number of recovered reads in metabarcoding datasets [36,37]. Importantly, because individual fecal samples are expected to originate from a single host individual, low frequency reads assigned to non-dominant lineages are more appropriately interpreted as secondary signals rather than as evidence of biological co-occurrence within a sample. Accordingly, lineage identification should rely primarily on the dominant assignment per sample, whereas read proportions across the dataset should not be interpreted as direct estimates of relative lineage abundance without experimental validation of primer performance across *P. chilensis*, *P. andinus* and *P. jamesi*.

Fresh fecal samples generally preserve higher-quality host-derived DNA, improving the reliability of species identification and supporting potential downstream applications such as mitochondrial haplotyping or population genetic analyses based on non-invasive material [38]. In contrast, environmentally exposed or dry feces are more susceptible to DNA fragmentation, oxidation, ultraviolet degradation, and environmental contamination, potentially reducing host genotyping accuracy and increasing stochastic amplification effects. These limitations are particularly relevant when attempting to discriminate closely related or sympatric taxa using degraded mitochondrial DNA. Similar effects have been described in fecal metagenomic studies, where sample preservation and environmental exposure substantially influence DNA recovery efficiency and downstream genomic inference [38,39].

Beyond host DNA, fecal condition may also affect microbiome characterization and dietary metabarcoding analyses. Fresh fecal samples are more likely to preserve microbial communities representative of the host intestinal microbiota, whereas dry or environmentally exposed feces may undergo post-depositional microbial succession and environmental colonization, potentially altering microbial composition profiles [39,40]. Likewise, dietary metabarcoding analyses can be affected by differential degradation of prey-derived DNA, leading to biased recovery of consumed taxa under adverse environmental conditions [41]. Therefore, wet or freshly deposited fecal material is expected to provide more reliable information for simultaneous host identification, microbiome characterization, and trophic ecology reconstruction in non-invasive metabarcoding studies.

The recovery of lineage-informative ASVs indicates that the selected mitochondrial marker captured sufficient variation to distinguish *Phoenicopterus* from Parina-associated lineages. ASV-based analysis was particularly useful because it retains exact sequence variants after error correction, improving resolution when closely related species or lineages are analyzed. The *Phoenicopterus* ASVs were almost exclusively recovered from Zoológico Nacional samples, in agreement with their expected *P. chilensis* origin, whereas samples from Laguna de Machuca were associated with both Parina lineages. This distribution indicates that the recovered ASVs followed a biologically meaningful pattern related to sampling locality and expected flamingo assemblages.

The separation between Parina lineage A and Parina lineage B suggests the presence of at least two genetically differentiated Parina-associated lineages in the analyzed fecal samples. However, current reference limitations for *P. andinus* and *P. jamesi* prevent definitive species-level assignment. Therefore, the use of operational lineage names is appropriate as a conservative taxonomic decision, avoiding assignments beyond the resolution supported by available reference data.

The hierarchical probabilistic framework provided a robust strategy for assigning fecal samples while accounting for low-frequency secondary reads. Metabarcoding datasets commonly contain minor reads derived from sequencing noise, index hopping, low-level carryover, environmental trace DNA, or true biological mixtures [42,43]. The agreement between maximum likelihood and Bayesian Dirichlet models supports the reliability of the assignments, as all samples were assigned to a dominant lineage with high probability and none was classified as mixed, ambiguous, or low-information [44]. Minor secondary signals, such as the low Parina B component detected in LM-F05 and ZN-F12, were far below the dominant lineage signal and are more parsimoniously interpreted as technical or environmental background. From a practical perspective, assignment thresholds should be interpreted together with total informative read depth, dominant-lineage probability, and the absolute number of secondary reads. Low-depth samples and samples with dominant-lineage probabilities close to 0.95 require additional caution because small changes in read counts can have a disproportionate effect on estimated proportions. We therefore recommend that future applications explicitly flag borderline or low-information samples and report total informative read depth together with dominant and secondary lineage probabilities. This practice would allow users to distinguish highly robust classifications from cases requiring confirmatory sequencing, replicate analysis, or additional ecological evidence. In conservative applications aimed at single-host assignment, secondary signals below 1–3% of sample-level informative reads, or below 5% under a more stringent criterion, should generally be treated as probable background unless they are reproducible across replicates or supported by independent biological evidence.

Low-abundance reads from non-dominant lineages are commonly reported in high-throughput metabarcoding studies and may arise from several technical processes, including index misassignment or sample cross-talk, tag-jumping during library preparation, stochastic PCR effects, or low-level carryover contamination. Previous studies have shown that sample cross-talk and index misassignment can generate rare sequences incorrectly assigned to samples, particularly when large numbers of multiplexed libraries are sequenced simultaneously [45]. Likewise, tag-jumping events have been identified as an important source of low-frequency false-positive detections in metabarcoding datasets [46,47]. Because the present analyses were based on fecal samples, which are expected to be dominated by the DNA of a single host lineage, minor cross-lineage reads were interpreted conservatively and were not considered primary evidence of true lineage co-occurrence. This interpretation is also consistent with the probabilistic assignments, in which each sample showed a dominant lineage signal and no sample was classified as mixed or ambiguous.

NGS-based fecal identification could provide a useful complementary tool for flamingo monitoring in northern Chile and other high-Andean wetland systems. Traditional monitoring relies heavily on visual surveys, which are essential but can be limited by accessibility, distance, mixed-species flocks, observer experience, and temporal variation in habitat use. Fecal DNA provides a complementary, non-invasive source of information that can confirm species or lineage presence without capturing, handling, or disturbing birds. The detection of both Parina lineage A and Parina lineage B in Laguna de Machuca demonstrates how this approach can reveal genetic heterogeneity that would be difficult to infer from fecal morphology alone. Beyond host identification, fecal metabarcoding could also provide information on diet, trophic interactions, and local biodiversity when appropriate dietary markers and reference databases are available. This is relevant for flamingos because feeding ecology can vary among species, habitats, and seasons. Thus, integrating host identification with dietary metabarcoding would allow researchers to determine which flamingo lineage used a site and what biological resources were consumed.

A directly comparable study by Sacco et al. (2025) [15] recently demonstrated the applicability of multi-assay eDNA metabarcoding for detecting metazoan diversity in Chilean high-altitude hypersaline lakes using water and sediment samples. Their study detected a broad range of taxa, including aquatic invertebrates, endemic gastropods, and flamingos, and showed that eDNA can complement conventional biodiversity surveys in remote saline wetlands. Importantly, Sacco et al. also reported the detection of *Phoenicopterus* across sampled sites, while *P. andinus* and *P. jamesi* were not resolved, likely due in part to limited reference sequence availability for this group. This limitation is consistent with the main constraint identified in our study: the incomplete reference framework for South American flamingos. In this context, our fecal metabarcoding framework provides a complementary approach focused on host-associated DNA from non-invasive samples, while future integration with improved reference mitogenomes and dietary markers could extend its use to species-level identification, diet reconstruction, and trophic ecology in sympatric flamingo assemblages.

Although the newly assembled *P. chilensis* mitogenome improves the reference base for this species, definitive assignment of Parina lineage A and Parina lineage B will require complete mitogenomes and validated marker sequences from *P. andinus* and *P. jamesi*. Such improvements would allow the operational Parina lineages recovered here to be assigned to named species with greater confidence, particularly in high-Andean wetlands where both species may occur sympatrically and where visual identification or fecal morphology alone may be insufficient. Broader sampling across additional wetlands, seasons, and years will also be required to evaluate whether these lineages reflect recurrent site use, local abundance patterns, breeding activity, or seasonal movement dynamics.

Other methodological considerations should also be noted. Mitochondrial markers represent a single maternally inherited genomic compartment and do not capture nuclear genomic variation, hybridization, or finer population structure [48,49]. Primer bias may also influence read abundance among taxa; therefore, read proportions should be interpreted primarily as evidence of dominant host-associated signal rather than as quantitative estimates of lineage abundance [37]. Future applications would benefit from complementary nuclear markers, technical replicates, and validation assays including reference material from all three South American flamingo species.

Overall, this study demonstrates that mitogenome-informed metabarcoding can support the non-invasive identification of flamingo fecal samples in mixed-species systems. By generating a *P. chilensis* mitogenome and linking it to marker evaluation and fecal DNA sequencing at ASV-level resolution, this work provides a reference-based framework for distinguishing Chilean flamingo-associated DNA from Parina-associated lineages. As reference databases improve, this strategy could be refined to assign Parina lineages to *P. andinus* or *P. jamesi* with greater confidence, and applied to biodiversity monitoring, conservation planning, habitat-use studies, and environmental impact assessment in high-Andean aquatic ecosystems.

## 5. Conclusions

This study developed a mitogenome-informed metabarcoding framework for the non-invasive molecular identification of flamingo fecal samples collected in northern Chile. By generating the complete mitochondrial genome of *P. chilensis*, we established a species-specific reference for evaluating metabarcoding markers and interpreting short fecal DNA sequences. The approach successfully recovered flamingo-associated ASVs and distinguished *Phoenicopterus* from two Parina-associated genetic lineages using ASV-level resolution and probabilistic taxonomic assignment. Samples from the Zoológico Nacional were consistently assigned to *Phoenicopterus*, whereas samples from Laguna de Machuca revealed two operational Parina lineages. These results show that fecal DNA can provide reliable, non-invasive evidence of flamingo lineage presence in mixed-species wetland systems. Overall, the framework integrates mitogenomic resources, high-throughput sequencing, and statistical assignment, offering a useful tool for biodiversity monitoring, conservation planning, habitat-use studies, and environmental assessment in fragile high-Andean ecosystems.

The principal remaining constraint is the incomplete reference database for South American flamingos, which currently prevents definitive species-level assignment of Parina lineage A and Parina lineage B to *P. andinus* or *P. jamesi*. Future work should incorporate complete mitogenomes, validated marker sequences and reference tissue material for these species, expand sampling across wetlands, seasons, and years, and integrate dietary metabarcoding markers to link host identity with diet and trophic ecology. These improvements would allow the proposed framework to move from lineage-level assignment toward more precise species-level monitoring of sympatric flamingo assemblages.

## Figures and Tables

**Table 1:** Geographic locations, coordinates, and sampling dates of fecal samples analyzed in this study.

Sample Location	Coordinates	Administrative Region	Sampling Date	Number of Samples
Laguna de Machuca	22.612782° S, 68.056413° W	Antofagasta Region	4 August 2025	8
Zoológico Nacional	33.429722° S, 70.634167° W	Metropolitan Region	8 August 2025	9

**Table 2:** Primer sequences used for Sanger-based amplification of the mitochondrial 12S region in *P. chilensis*.

Primer	Sequence
MiBird-U-F	5′-GGGGTTGGTAAATCTTGTGCCAGC-3′
MiBird-U-R	5′-CATAGTGGGGTATCTAATCCCAGTTTG-3′

**Table 3:** Distribution of flamingo-associated ASV read counts across fecal samples.

ASVs	LM-F01	LM-F02	LM-F03	LM-F04	LM-F05	LM-F06	LM-F07	LM-F08	ZN-F01	ZN-F02	ZN-F03	ZN-F06	ZN-F07	ZN-F09	ZN-F10	ZN-F11	ZN-F12
Total informative reads	5443	5040	26,682	4729	8977	4780	5478	7082	5420	5326	4798	6071	5494	2898	1822	1555	1715
0.95 assignment threshold	5171	4788	25,348	4493	8529	4541	5205	6728	5149	5060	4559	5768	5220	2754	1731	1478	1630
0.05 secondary-signal threshold	272	252	1334	236	448	239	273	354	271	266	239	303	274	144	91	77	85
*Phoenicopterus* 01	0	0	0	0	0	0	0	0	5420	5321	4790	6071	5494	2898	7	1555	1705
*Phoenicopterus* 02	0	0	0	0	0	0	0	0	0	0	0	0	0	0	1815	0	0
Parina lineage A 01	5443	5040	0	4729	8923	4780	12	21	0	0	0	0	0	0	0	0	0
Parina lineage A 02	0	0	0	0	0	0	0	0	0	0	0	0	0	0	0	0	0
Parina lineage A 03	0	0	0	0	0	0	0	0	0	0	0	0	0	0	0	0	0
Parina lineage B 01	0	0	0	0	0	0	5466	7061	0	5	8	0	0	0	0	0	10
Parina lineage B 02	0	0	26,682	0	54	0	0	0	0	0	0	0	0	0	0	0	0

Threshold values were calculated from the total number of informative flamingo-associated reads per sample. The 0.95 threshold indicates the minimum number of reads required for conservative dominant-lineage assignment, whereas the 0.05 threshold indicates the maximum number of secondary-lineage reads compatible with a ≤5% background signal.

**Table 4:** Species-level assignment of fecal samples using maximum likelihood and Bayesian Dirichlet models. Samples were assigned to Parina lineage A, Parina lineage B, or *Phoenicopterus* according to the dominant posterior probability.

Sample	Method	Parina A	Parina B	*Phoenicopterus*	Assigned Species	Method	Parina A	Parina B	*Phoenicopterus*	Assigned Species
LM-F01	MLE	1.00	0.00	0.00	Parina A	Bayesian Dirichlet	1.000	0.000	0.000	Parina A
LM-F02	MLE	1.00	0.00	0.00	Parina A	Bayesian Dirichlet	1.000	0.000	0.000	Parina A
LM-F03	MLE	0.00	1.00	0.00	Parina B	Bayesian Dirichlet	0.000	1.000	0.000	Parina B
LM-F04	MLE	1.00	0.00	0.00	Parina A	Bayesian Dirichlet	1.000	0.000	0.000	Parina A
LM-F05	MLE	0.99	0.01	0.00	Parina A	Bayesian Dirichlet	0.994	0.006	0.000	Parina A
LM-F06	MLE	1.00	0.00	0.00	Parina A	Bayesian Dirichlet	1.000	0.000	0.000	Parina A
LM-F07	MLE	0.00	1.00	0.00	Parina B	Bayesian Dirichlet	0.002	0.998	0.000	Parina B
LM-F08	MLE	0.00	1.00	0.00	Parina B	Bayesian Dirichlet	0.003	0.997	0.000	Parina B
ZN-F01	MLE	0.00	0.00	1.00	*Phoenicopterus*	Bayesian Dirichlet	0.000	0.000	1.000	*Phoenicopterus*
ZN-F02	MLE	0.00	0.00	1.00	*Phoenicopterus*	Bayesian Dirichlet	0.000	0.001	0.999	*Phoenicopterus*
ZN-F03	MLE	0.00	0.00	1.00	*Phoenicopterus*	Bayesian Dirichlet	0.000	0.002	0.998	*Phoenicopterus*
ZN-F06	MLE	0.00	0.00	1.00	*Phoenicopterus*	Bayesian Dirichlet	0.000	0.000	1.000	*Phoenicopterus*
ZN-F07	MLE	0.00	0.00	1.00	*Phoenicopterus*	Bayesian Dirichlet	0.000	0.000	1.000	*Phoenicopterus*
ZN-F09	MLE	0.00	0.00	1.00	*Phoenicopterus*	Bayesian Dirichlet	0.000	0.000	1.000	*Phoenicopterus*
ZN-F10	MLE	0.00	0.00	1.00	*Phoenicopterus*	Bayesian Dirichlet	0.000	0.000	1.000	*Phoenicopterus*
ZN-F11	MLE	0.00	0.00	1.00	*Phoenicopterus*	Bayesian Dirichlet	0.000	0.000	1.000	*Phoenicopterus*
ZN-F12	MLE	0.00	0.01	0.99	*Phoenicopterus*	Bayesian Dirichlet	0.000	0.006	0.994	*Phoenicopterus*

## Data Availability

The complete mitochondrial genome of *Phoenicopterus chilensis* is available in GenBank under accession number PZ609876. The raw sequencing data are available from the corresponding authors upon reasonable request.

## Supplementary Materials

The following supporting information can be downloaded at: https://www.mdpi.com/article/10.3390/biology15151295/s1, Figure S1: Tissue sample of *P. chilensis* for mitochondrial DNA extraction. (A) Whole specimen showing general morphology and body size. (B) Dissection of the abdominal region indicating the anatomical location used for tissue collection. (C) Close-up of liver tissue extraction performed under sterile conditions. The obtained tissue was used for genomic DNA extraction and subsequent mitogenome assembly.

## Abbreviations

The following abbreviations are used in this manuscript:

Abbreviation	Full term
ASV	Amplicon sequence variant
COI	Cytochrome c oxidase subunit I
DNA	Deoxyribonucleic acid
eDNA	Environmental DNA
MLE	Maximum likelihood estimation
NGS	Next-generation sequencing
PCR	Polymerase chain reaction
rRNA	Ribosomal ribonucleic acid
SNP	Single nucleotide polymorphism
UCE	Ultraconserved element
